# Whole genome sequencing of *Saccharomyces cerevisiae*: from genotype to phenotype for improved metabolic engineering applications

**DOI:** 10.1186/1471-2164-11-723

**Published:** 2010-12-22

**Authors:** José Manuel Otero, Wanwipa Vongsangnak, Mohammad A Asadollahi, Roberto Olivares-Hernandes, Jérôme Maury, Laurent Farinelli, Loïc Barlocher, Magne Østerås, Michel Schalk, Anthony Clark, Jens Nielsen

**Affiliations:** 1 Department of Chemical and Biological Engineering, Chalmers University of Technology, SE-41296 Gothenburg, Sweden; 2 Center for Microbial Biotechnology, Department of Systems Biology, Technical University of Denmark DK-2800, Kgs. Lyngby, Denmark; 3 Fasteris SA, Geneva, Switzerland; 4 Firmenich SA, Corporate Research & Development Division, Geneva, Switzerland; 5 Vaccine & Biologics Process Development, Vaccine Research & Development, Merck Research Labs, West Point, PA, USA; 6 Biotechnology Group, Faculty of Advanced Sciences and Technologies, University of Isfahan, Isfahan 81746-73441, Iran; 7 Fluxome Sciencies A/S, Research & Development, DK-3660 Stenlose, Denmark; 8 Center for Systems Biology, Soochow University, Suzhou 215006, China

## Abstract

**Background:**

The need for rapid and efficient microbial cell factory design and construction are possible through the enabling technology, metabolic engineering, which is now being facilitated by systems biology approaches. Metabolic engineering is often complimented by directed evolution, where selective pressure is applied to a partially genetically engineered strain to confer a desirable phenotype. The exact genetic modification or resulting genotype that leads to the improved phenotype is often not identified or understood to enable further metabolic engineering.

**Results:**

In this work we performed whole genome high-throughput sequencing and annotation can be used to identify single nucleotide polymorphisms (SNPs) between *Saccharomyces cerevisiae *strains S288c and CEN.PK113-7D. The yeast strain S288c was the first eukaryote sequenced, serving as the reference genome for the *Saccharomyces *Genome Database, while CEN.PK113-7D is a preferred laboratory strain for industrial biotechnology research. A total of 13,787 high-quality SNPs were detected between both strains (reference strain: S288c). Considering only metabolic genes (782 of 5,596 annotated genes), a total of 219 metabolism specific SNPs are distributed across 158 metabolic genes, with 85 of the SNPs being nonsynonymous (e.g., encoding amino acid modifications). Amongst metabolic SNPs detected, there was pathway enrichment in the galactose uptake pathway (*GAL1*, *GAL10*) and ergosterol biosynthetic pathway (*ERG8*, *ERG9*). Physiological characterization confirmed a strong deficiency in galactose uptake and metabolism in S288c compared to CEN.PK113-7D, and similarly, ergosterol content in CEN.PK113-7D was significantly higher in both glucose and galactose supplemented cultivations compared to S288c. Furthermore, DNA microarray profiling of S288c and CEN.PK113-7D in both glucose and galactose batch cultures did not provide a clear hypothesis for major phenotypes observed, suggesting that genotype to phenotype correlations are manifested post-transcriptionally or post-translationally either through protein concentration and/or function.

**Conclusions:**

With an intensifying need for microbial cell factories that produce a wide array of target compounds, whole genome high-throughput sequencing and annotation for SNP detection can aid in better reducing and defining the metabolic landscape. This work demonstrates direct correlations between genotype and phenotype that provides clear and high-probability of success metabolic engineering targets. The genome sequence, annotation, and a SNP viewer of CEN.PK113-7D are deposited at http://www.sysbio.se/cenpk.

## Background

Metabolic engineering is the enabling technology for identification of targeted genetic modifications such as gene deletion, over-expression, or modulation. The genetic engineering implemented in a host microbial cell factory ideally will lead to re-direction of fluxes to enhance production or robustness of a given product or organism, respectively [1-5]. Metabolic engineering through systems biology has been complimented, and its application expanded in both scope and success. Systems biology is a multi-disciplinary approach to quantitative collection, analysis, and integration of whole genome scale data sets enabling construction of biologically relevant and often predictive mathematical models [6-8]. Genome sequencing of industrially relevant organisms, including *S. cerevisiae *strain S288c, the first eukaryote genome sequence reported, provided a framework for gene annotation through functional genomics. More relevant to metabolic engineering, an annotated genome sequence was a prerequisite for genome-scale metabolic network reconstructions [9,10]. Such reconstructions offer a biochemical model describing the formation and depletion of each metabolite that by providing mass-balance boundary conditions makes possible constraint based simulations of how the metabolic network operates at different conditions. In simpler terms, using basic stoichiometry these models can be used to predict the relationships between gene functions in the cellular metabolic network. With nearly 14 years elapsing since the *S. cerevisiae *strain S288c genome sequence was made available, and more than 1,000 laboratories participating in functional genomics efforts, there are still 968 and 811 open reading frames (ORFs) classified as uncharacterized and dubious, respectively, according to the *Saccharomyces *Genome Database (SGD) [10,11]. Furthermore, since 2003 there have been published five major *S. cerevisiae *genome-scale metabolic network reconstructions, with the most recent models encompassing between 13-14% genome coverage [12,13]. The opportunity to further extend genotype to phenotype annotation is abundant.

Industrial biotechnology is dominated by efforts to confer a desirable phenotype onto strains using different methods of directed evolution and random mutagenesis, requiring screening and selection. This approach, while providing little to no mechanistic understanding of which specific genetic perturbations lead to improved strains so they could be further exploited, has proven to be commercially successful as illustrated by the more than 1,000 fold improvement in penicillin titer by *Penicillium chrysogenum *[14]. As industrial biotechnology applications expand, and the desire to custom-engineer microbial cell factories with novel architecture for native and heterologous metabolic pathways increases, the necessity on a genome-wide level to understand direct genotype to phenotype relationships has rapidly increased. Within the same time period of approximately the last 10 years, the technologies and costs associated with whole genome sequencing have advanced and decreased, respectively. There are several excellent reviews of genome sequencing technologies, and their applications to functional genomics, strain engineering, and other investigatory biology efforts [5,15-18]. Prior work, specifically focused on characterizing genome-wide analysis of nucleotide polymorphisms in *S. cerevisiae *utilized 25mers oligonucleotide microarrays (Affymetrix yeast tiling arrays) providing random and redundant coverage of the *S. cerevisiae *genome [19]. This analysis included single nucleotide polymorphism (SNP) identification between S288c and the commonly used laboratory strain *S. cerevisiae *CEN.PK, where a total of 13,914 SNPs were identified. However, this approach is unable to identify the exact nucleotide substitution, and consequently whether the transcribed SNP results in an amino acid substitution, presumably required to confer a change in enzyme and/or protein function. More recently a collaborative project, the *Saccharomyces *Genome Resequencing Project (SGRP) between the Sanger Institute and Institute of Genetics, University of Nottingham, completed the ABI sequencing of haploids of 37 *S. cerevisiae *strains to a coverage of 1-3X. Furthermore, Illumina-Solexa genome sequencing of four of the 37 *S. cerevisiae *strains, one of which included S288c, was completed [20]. This sequencing effort was focused on exploration of genomic variation in the context of evolution, thereby using multiple strains from different *Saccharomyces *species. It is a demonstration of a recent genome sequencing technology, referred to as Illumina-Solexa sequencing, compared to larger read methods such as Sanger or 454 sequencing. Illumina-Solexa sequencing is an ultra-high-throughput technology that performs sequencing-by-synthesis of random arrays of clonal DNA colonies attached to the surface of a flow-cell. The approach used in this study generates short, 35 base pair (bp) reads (currently, the technology limitations are 150 bp or 2 × 150 bp paired-reads), that must then be aligned to and assembled using a reference genome [21-23].

In this work we propose that high-throughput genome sequencing and annotation, integrated with a genome browser and SNP viewer of *S. cerevisiae *may serve as a commonplace tool, complementary to transcriptomics and physiological characterization, to extract direct genotype to phenotype information. Firstly, whole genome Illumina-Solexa sequencing of each strain was completed and then annotation was performed. To access annotated genome sequences and detected SNPs of CEN.PK113-7D, a genome browser and SNP viewer were developed. SNPs strictly related to metabolic genes were identified, characterized, and amino acid level analysis performed. In order to directly link genotype to phenotype cellular behavior was characterized in well-controlled batch fermentations on glucose and galactose, complimented with transcriptome analysis. More specifically, we demonstrate that S288c, the strain utilized for the publically available *S. cerevisiae *genome sequence, exhibits atypical *S. cerevisiae *behavior related to central carbon metabolism as compared to CEN.PK113-7D, a common laboratory strain for industrial biotechnology applications [24].

There were clear correlations between physiology and metabolic pathway enrichment of nonsynonymous SNPs observed, suggesting that sequencing and the annotated genome may assist in reducing the genetic target space for metabolic engineering applications. The analysis presented here serves as a foundation for comparative metabolic engineering SNP analysis, wherein the future reference strains may be compared to their metabolically engineered derivatives that use directed evolution in order to answer the age-old question: what changed in our strain that makes it a preferred microbial cell factory?

## Results

### Genome Sequencing and Annotation

The genome sequencing of *S. cerevisiae *strain CEN.PK113-7D and genome re-sequencing of strain S288c were accomplished using the Illumina/Solexa technology. According to the manufacturer's recommendations (Illumina), raw sequence reads of strain CEN.PK113-7D and strain S288c were accumulated to approximately 18× and 15× depth coverage, respectively. Reads were mapped on the S288c as reference genome using MAQ software http://maq.sourceforge.net. Based on genome sequence analysis and annotation (See Methods), the 12.1 Mb genome was predicted to contain a total of 5,596 genes encoding proteins. The genome was predicted to comprise 16 chromosomes by pair-wise comparison against the 16 different chromosomes of the public reference strain S288c from the *Saccharomyces *Genome Database (SGD). Interestingly, we found that genome characteristics of strain CEN.PK113-7D are very similar to S288c for genome parameters that include total size, chromosome length, GC content, and the number of predicted genes. Table 1 summarizes Illumina genome sequencing and annotation results.

**Table 1:** Illumina/Solexa genome sequencing and annotation results

Sequencing Parameter	S288c	CEN.PK113-7D
**No. of Reads**	5,301,907	6,603,200
**No. of Aligned Reads**	5,176,155	6,216,656
**Total Bases^A ^(bp)**	181,165,425	217,582,960
**Calculated Average Coverage**	15x	18×
**Genome Percent Reference Coverage (%)**	99.9	99.4
**No. of chromosomes**	16	16
**%GC content**	38.3	38.3

**Notes: **Base pairs (bp). (A) Each read is 35 base pairs in length.

### Genome Browser and SNP Viewer

**Figure 1:**
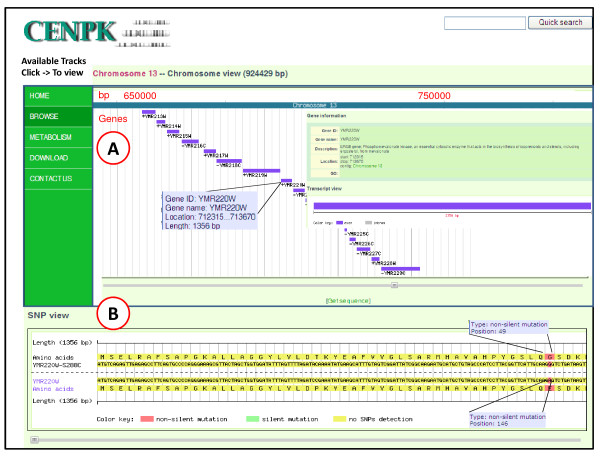
**Screenshot of Genome Browser and SNP viewer displaying chromosome 13 of *S. cerevisiae *CEN.PK113-7D**. The navigation panel (A) includes an overview of the genome, gene ID, gene name, gene function and the current location in the chromosome, together with exon and intron structure, and a text box for quick searching. Below this is a frameshowing the SNP viewer. (B) SNP information section for displaying nucleotide and amino acid polymorphism data between yeast *S. cerevisiae *strains.

To visualize genome sequences, annotated genes, and detected SNPs of *S. cerevisiae *CEN.PK 113-7D, a PHP-MySQL-based genome browser and SNP viewer were developed and deposited on the web-site (See http://www.sysbio.se/cenpk). The basic genome browser functionality, as seen in Figure 1, can provide genome annotation views with an overhead bar providing a visual indication of chromosome position. It is possible to navigate by dragging the display left or right creating a smooth panning effect. Alternatively, one can navigate directly to a region (e.g., gene name) of interest by providing the region coordinates or typing a feature name into the quick search box. The browser can display basic genomic features of interest (e.g., geneID, gene name, gene function, location, gene ontology process, and exon/intron structures). Similarly, for the SNP viewer one can visualize nucleotide and amino acid polymorphism data between *S. cerevisiae *strains. The whole gene containing a SNP (s) can be displayed by dragging left or right button (which creates a smooth panning effect), and also highlight the mutated position (e.g., nonsynonymous SNP (s), silent SNP (s)) between *S. cerevisiae *strains.

### SNPs identification in metabolism

Based on whole genome sequencing, data that includes the number of reads, average coverage relative to the SGD reference genome, total number of non-ambiguous SNPs, and total number of filtered SNPs and detected SNPs error are presented in Table 2. Not surprisingly, S288c had relatively few SNPs compared to CEN.PK113-7D given that the reference genome from SGD is based on S288c version 12.0 [10,11]. Furthermore, the 13,787 filtered SNPs identified using the MAQ software is consistent with the previously estimated 13,914 SNPs for CEN.PK113-7D based upon DNA hybridization to 25mers olignonucleotide microarrays [19]. Table 2 also presents the results for metabolic SNP detection, where a total of 782 metabolic genes as defined by SGD were used to query for SNPs in both the S288c and CEN.PK113-7D genome sequences. A total of 36 metabolic SNPc of S288c, 3 of which are nonsynonymous, were identified across 14 independent metabolic genes (3 nonsynonymous SNPs distributed across 3 metabolic genes). A significantly higher number of metabolic SNPs, 939, were detected in CEN.PK113-7D and distributed across 158 unique metabolic genes, 85 of which contained a total 219 nonsynonymous SNPs.

**Table 2:** Genome sequencing and metabolic SNP detection

*Sequencing Parameter*	S288c	CEN.PK113-7D
***MAQ Software Determination***		

**No. of Reads**	5,301,907	6,603,200
**Total No. of SNPs**	3,032	27,868
**Total No. of Non-Ambiguous SNPs**	1,013	24,663
**Total No. of Filtered SNPs^A,B^**	311	13,787

***Metabolism Focused Detection***		

**Total No. of Metabolic Genes Considered^C^**	782	782
**Total No. of Metabolic Bases (bp)**	1.16M	1.16M
**No. of Aligned Reads**	477,565	623,400
**Total Gap Size (bp)**	0	0
**Metabolic Genome Percent Reference Coverage (%)**	99.7	99.4
**Total No. of Metabolic SNPs detected**	36	939
**Total No. Nonsynonymous Metabolic SNPs Detected**	3	219
**Percent of SNPs Detected Nonsynonymous (%)**	8.3	23.3
**Total No. of Metabolic Genes Containing SNP**	14	158
**Total No. of Metabolic Genes with Nonsynonymous SNP**	3	85

**Notes: **Base pairs (bp). (A) Filtered SNPs determined based on cut-off criteria within the Mapping and Assembling with Quality (MAQ) software environment. (B) Detected SNPs with estimated error < 0.003% (C) The total number of genes classified as metabolic was based on the *Saccharomyces *Genome Database, Strain S288c, version 12.0. The "S288c" designation in Table 2 refers to the resequencing of *S. cerevisiae *S288c using Illumnia/Solexa sequencing technology.

**Figure 2:**
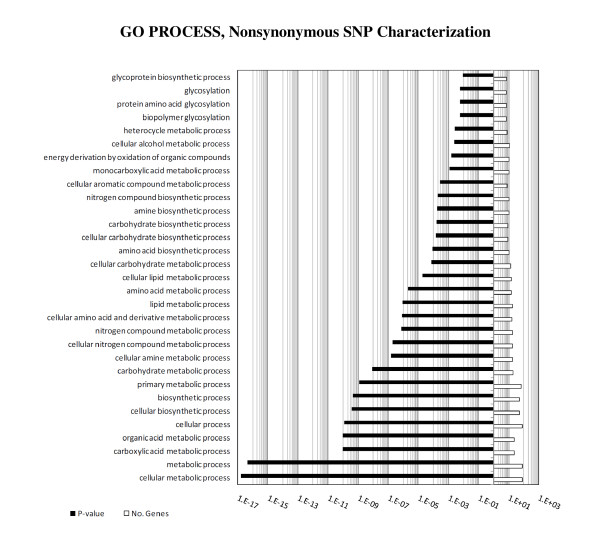
**Gene Ontology (GO) process terms for the nonsynonymous SNPs identified in CEN.PK113-7D compared to S288c**. The *x*-axis in log-scale displays both the significance of each category (*p *< 0.01, symbol: solid black), and the number of genes from the total of 85 containing nonsynonymous SNPs (symbol: solid white). GO process characterization performed using the *Saccharomyces *Genome Database (SGD).

In an effort to characterize the nonsynonymous metabolic SNPs identified in CEN.PK113-7D with biological significance, Gene Ontology (GO) process categorization was performed and presented in Figure 2 ranked according to statistical significance (*p *< 0.01). The most significant categories include carboxylic acid, organic acid, and carbohydrate metabolism, followed by nitrogen, amino acid, lipid, aromatic compound, and glycoprotein metabolism. Additional file 1 Figure S1 presents the GO function and component categorization, and as expected the highest significant concentration of nonsynonymous SNPs (*p *< 0.01) distributed across a specific enzyme class is for transferases. Notably, the background genes used for GO enrichment analysis were the complete gene set available in SGD (verified, unverified, and dubious). GO enrichment analysis uses a hypergeometric distribution with Multiple Hypothesis Correct to calculate *p*-values and correct for multiple sampling.

**Figure 3:**
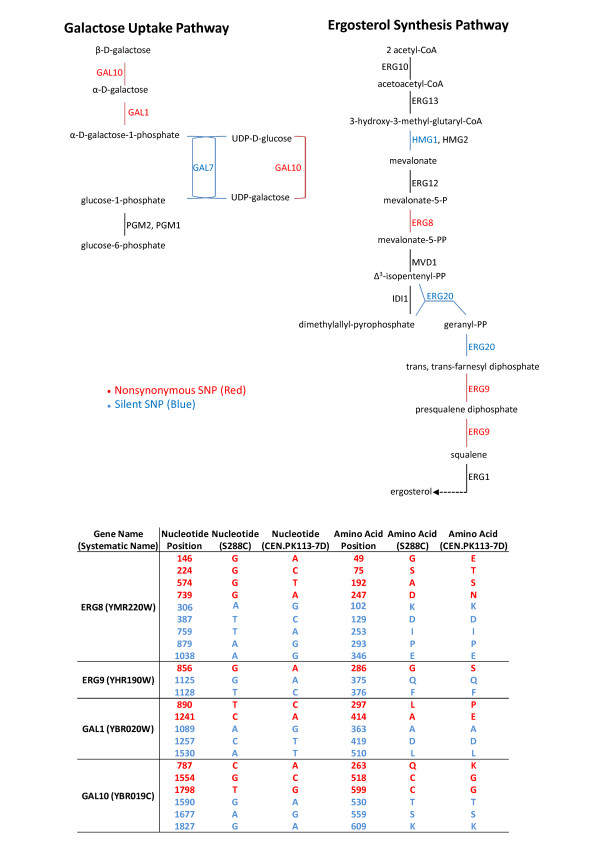
**Enriched SNP in galactose uptake pathway and ergosterol synthesis pathway**. Two pathways with a significant number of SNPs, both silent (blue) and nonsynonymous (red) are included: galactose uptake pathway and ergosterol synthesis pathway. The significance of each pathway (*p *< 0.01). Both standard single letter codes for nucleotides and amino acids are utilized.

A graphical representation of all silent and nonsynonymous SNPs mapped to their specific metabolic pathways is presented in Additional file 1 Figure S2. Figure 3 highlights two metabolic pathways, galactose uptake and ergosterol synthesis, where an enrichment of nonsynonymous and silent SNPs was observed. Specifically, *GAL1*, *GAL10*, *ERG8*, and *ERG9 *contained nonsynonymous SNPs, while *GAL7, ERG20 *and *HMG1 *contained silent SNPs. The specific SNPs are identified as well the resulting amino acid substitutions.

**Figure 4:**
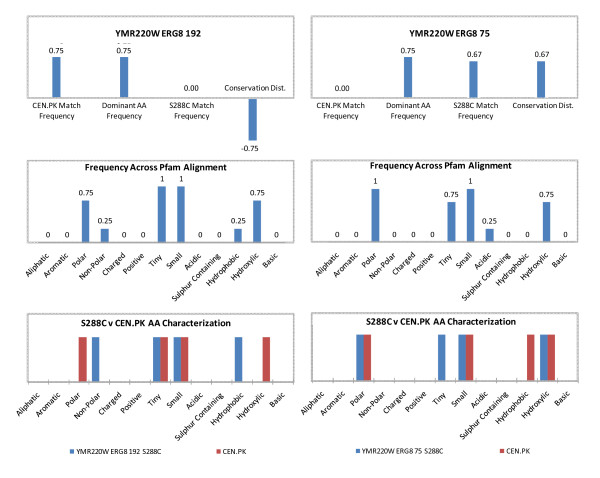
**An example of amino acid properties change of CEN. PK113-7D compared to S288c: the gene ERG8 of the ergosterol synthesis pathway**. The gene *ERG8 *of the ergosterol synthesis pathway contains a total of 4 nonsynonymous SNPs, two of which, located at nucleotide positions 192 and 75, are analyzed here. The top plots show the CEN.PK Match Frequency, Dominanat AA Frequency, S288c Match Frequency, and Conversation Distance. The middle plots show the frequency (fraction) of each categorization across the amino acid sequences resulting from Pfam multi-sequence alignment. The bottom plots shows the characterization of the original S288c amino acid (symbol: blue bar) and the CEN.PK113-7D amino acid (symbol: red bar). The gene *ERG8 *contained a total of 4 nonsynonymous SNPs, and Additional file 1, Figure S5 includes the other 2 nonsynonymous SNPs (nucleotide positions 49 and 247).

In addition to identifying SNP enriched metabolic pathways in CEN.PK113-7D, an analysis intended to determine the prevalence of the SNP across the top 10 homologous sequences resulting from a multi-alignment Pfam query was performed. To better quantify those results, the parameters *CEN.PK Match Frequency, Dominant AA Frequency, S288c Match Frequency*, and *Conservation Distance *were defined and calculated (See Additional file 1 Figure S3). The *Conservation Distance*, bound between -1 and 1, is a measure of whether the SNP identified in CEN.PK113-7D is more prevalent amongst homologous Pfam sequences (maximum Conservation Distance = -1), or if S288c (reference SGD sequence) is more prevalent (maximum Conservation Distance = +1). Additional file 1 Figure S4 presents the Conservation Distance across nonsynonymous SNPs identified, with the average value of 0.03 ± 0.40 (*n = *219), indicating that there is virtually no bias between S288c or CEN.PK113-7D as compared to their homologues. Extending this approach further, each amino acid polymorphism was characterized across a multi-alignment Pfam homologue search, and categorized according to standard amino acid properties (See Additional file 1 Figure S3). For example, Figure 4 presents SNPs identified in *ERG8 *at nucleotide positions 75 and 192. The resulting amino acid partially encoded by position 192 was 75% polar, 25% non-polar, 25% hydrophobic, and 75% hydroxylic looking across the top 10 Pfam homologous sequences. Lastly, and of most relevance to understanding the amino acid functional changes resulting from a SNP, the same categorization is presented for the S288c v. CEN.PK113-7D sequence. For example, the SNP at position 192 of *ERG8 *resulted in changing the encoded amino acid from non-polar (S288c) to polar (CEN.PK113-7D), and from hydrophobic (S288c) to hydroxylic (CEN.PK113-7D). This approach is extended to all the *ERG8 *nonsynonymous SNPs as an example of extending nucleotide level changes to amino acid functional changes (See Additional file 1 Figure S5 for additional *ERG8 *nonsynonymous SNPs). Furthermore, Additional file 1 Figure S6 highlights functional changes for all metabolic nonsynonymous SNPs identified.

### Physiological Characterization

**Figure 5:**
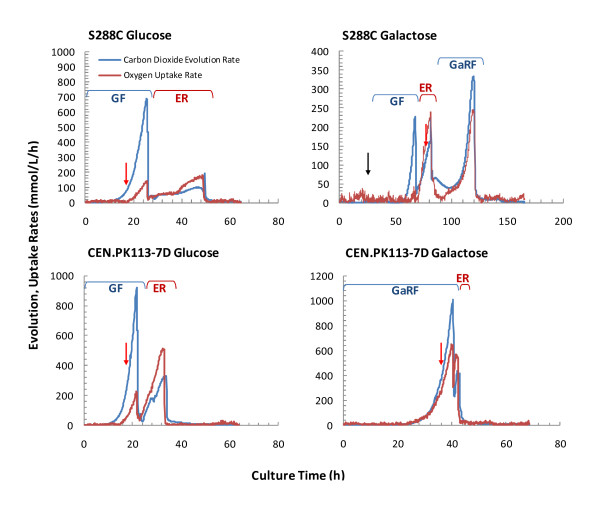
**Physiological characterization of *S. cerevisiae *S288c and CEN.PK113-7D**. The plots above show the carbon dioxide evolution rate and oxygen uptake rate as a function of cultivation time for the strains S288c and CEN.PK113-7D supplemented with glucose and galactose, respectively. Glucose fermentation (GF), ethanol respiration (ER), galactose respiro-fermentation (GaRF). The black arrow in the S288c Galactose plot indicates when 10 g L^-1 ^glucose was supplemented (25 h) when no growth was observed on galactose. The red arrows in all plots indicates when biomass samples were taken for subsequent transcriptome analysis.

The S*. cerevisiae *strains S288c and CEN.PK113-7D were physiologically characterized in both batch glucose and galactose supplemented fermentations. On glucose, CEN.PK113-7D exhibited a 32% higher specific growth rate than S288c, correlating with the 33% higher specific glucose consumption rate (See Table 3). The CEN.PK113-7D extracellular metabolic specific productivity rates were 32.6%, 392%, and 17.9% higher for ethanol, acetate, and glycerol production compared to S288c, respectively, while the specific oxygen consumption rates were nearly equivalent (1.98 O_2_-mmol g-DCW^-1 ^h^-1 ^for CEN.PK113-7D v. 1.95 mmol-O_2 _g-DCW^-1 ^h^-1 ^for S288c). Following complete glucose fermentation, as indicated by the peak carbon dioxide evolution rate (CER), both strains underwent a diauxic shift, clearly identified by the transition of the respiratory quotient (RQ) from > 1 to < 1, and ethanol accumulated during glucose fermentation (11.1 g L^-1 ^for CEN.PK113-7D v. 11.3 g L^-1 ^for S288c) was respiro-fermented. The ethanol respiro-fermentation (ERF) phase (Figure 5) was clearly distinguishable in the CEN.PK113-7D compared to S288c, where both CER and oxygen uptake rates (OUR) linearly increased, corresponding with the increase in biomass (3.7 to 12.0 g-DCW L^-1^). On the contrary, during the ERF phase for S288c there was a growth deficiency, clearly indicated by non-linear and significantly reduced CER and OUR rates, corresponding with a much lower increase in biomass (2.1 to 6.9 g-DCW L^-1^). The significantly decreased ERF phase in S288c compared to CEN.PK113-7D is also evident from the total time required to exhaust the ethanol (50 v. 33 h, respectively).

**Table 3:** Physiological characterization of *S. cerevisiae* strains S288c and CEN.PK113-7D

Strain	S288c		CEN.PK 113-7D		S288c		S288c		CEN.PK 113-7D	
**Substrate**	**Glucose**		**Glucose**		**Galactose**		**Galactose/** **Ethanol**		**Galactose**	

	**Mean**	***± SD*** ***(n = 2)***	**Mean**	***± SD*** ***(n = 2)***	**Mean**	***± SD*** ***(n = 2)***	**Mean**	***± SD*** ***(n = 2)***	**Mean**	***± SD*** ***(n = 2)***

**μ-max (h^-1^)**	0.31	0.01	0.41	0.01	0.02	0.00	0.14	0.01	0.27	0.00
**Carbon Recovery (%)**	96.60	1.90	95.50	3.90	n/a	n/a	n/a	n/a	79.60	2.60
**Specific Productivity or Consumption Rate^A^**										
**-r_Gluc _or -r_Gal_**	79.35	5.48	105.15	0.24	1.21	0.57	4.50	0.70	24.28	0.33
**r_CO2_**	18.36	0.52	23.62	0.87	0.11	0.01	0.58	0.12	4.31	0.24
**r_EtOH_**	37.59	4.39	49.88	0.34	0.01	0.01	-3.97	0.62	3.50	0.26
**r_Acet_**	0.24	0.02	1.18	0.04	0.00	0.00	-0.04	0.02	1.10	0.11
**r_Glyc_**	6.08	0.94	7.17	2.64	0.00	0.00	-0.58	0.05	0.89	0.09
**r_Pyr_**	0.47	0.04	0.69	0.06	0.00	0.00	0.00	0.00	0.04	0.01
**r_Suc_**	0.01	0.01	0.03	0.05	0.00	0.00	0.00	0.00	0.00	0.00
**r_X_**	13.85	1.11	17.79	0.02	0.78	0.05	5.85	0.02	9.49	0.89
**-r_O2_**	1.95	0.07	1.98	2.75	0.08	0.01	0.97	0.02	2.91	0.22

Notes: A. (C-mmol g-DCW^-1 ^h^-1^). The term "n/a" refers to not applicable.

A similar characterization was performed using batch galactose supplemented fermentations. CEN.PK113-7D demonstrated a slight lag-phase compared to glucose fermentation; however, sustained a galactose specific growth rate of 0.27 h^-1 ^and galactose uptake rate of 24.3 C-mmol g-DCW^-1 ^h^-1^, representing a 34% and 77% reduction, respectively, compared to glucose (See Table 3). All extracellular metabolic specific productivity rates were significantly decreased (ethanol, acetate, and glycerol were 93%, 6.8%, and 88% reduced compared to glucose, respectively), with the exception of OUR, which was 47% higher on galactose compared to glucose, leading to an effectively lower RQ of 1.5 compared to 11.9 during glucose cultivation. Furthermore, given the significantly lower RQ during the exponential phase of galactose fermentation, relatively little ethanol was produced (2.7 g L^-1^), resulting in a short ERF phase (< 5h) (See Figure 5). Similarly, S288c was cultivated on galactose; however, a significant deficiency in the strain's ability to metabolize this carbon source was observed. A total of 25 h post-inoculation elapsed with no increase in biomass as compared to CEN.PK113-7D where after 6 h post-inoculation two cell doublings were observed. At 25 h post-inoculation a glucose bolus of 10 g L^-1 ^was added to promote growth, and rapidly, glucose fermentation, a diauxic shift, and ethanol respiro-fermentation were observed (See Figure 5). Both co-consumption of galactose and ethanol, and a galactose only respiro-fermentative (GaRF) growth phase were observed. During co-consumption the specific growth rate was 0.14 h^-1^, while on galactose only the specific growth rate was 0.02 h^-1^. Similarly, the extracellular specific metabolite productivity rates were nearly zero when only galactose consumption was considered (See Table 3). Ethanol was consumed by 82 h post-inoculation, and in the period from 82 h to 128 h, only galactose consumption was observed, and biomass increased from 7.9 g-DCWL L^-1 ^to 20.9 g-DCW L^-1^, representing a doubling time of 35 h compared to 2.6 h for CEN.PK113-7D.

**Figure 6:**
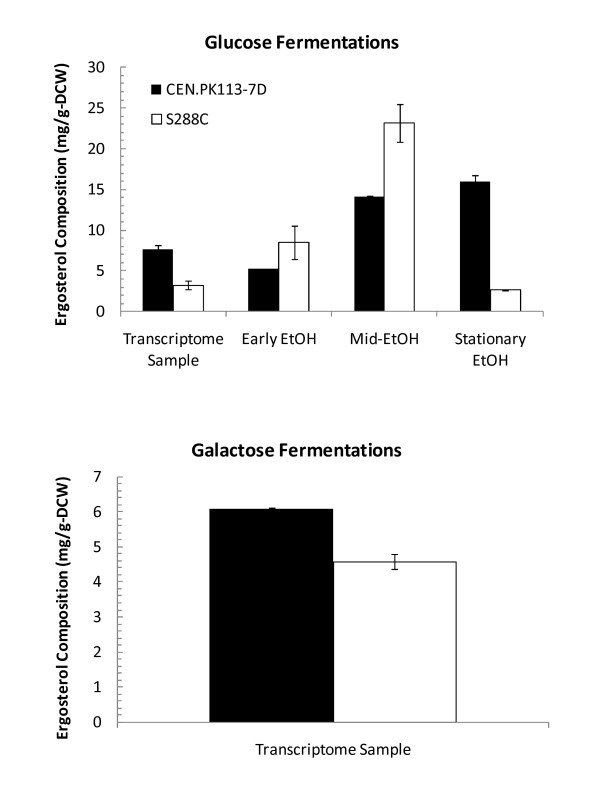
**Ergosterol measurements in *S. cerevisiae *strains S288c and CEN.PK113-7D**. Ergosterol composition (mg g-DCW^-1^) was measured for different samples taken during S288c and CEN.PK113-7D fermentations, supplemented with glucose and galactose. Transcriptome sample was taken during the mid-exponential fermentation phase on glucose or respiration phase on galactose. For glucose fermentations, early ethanol, mid-ethanol, and stationary ethanol samples were taken post-diauxic shift to charcterize the change in ergosterol during growth on ethanol. Error bars are ± SD (*n *= 2).

For each cultivation condition and strain, ergosterol measurements were performed and presented in Figure 6. At the same time of transcriptome sampling, which occurred during mid-exponential phase of glucose fermentation (18-20 h), a total ergosterol of 7.6 ± 0.5 mg g-DCW^-1 ^and 3.3 ± 0.5 mg g-DCW^-1 ^for CEN.PK113-7D and S288c, respectively, was measured. Subsequently, the diauxic shift and ERF phase was characterized by two ergosterol samples during early and mid-ERF phase, and followed by a final (stationary) sample post-ethanol exhaustion. S288c ergosterol content was significantly higher during ethanol metabolism as compared to CEN.PK113-7D, but post-ethanol metabolism CEN.PK113-7D exhibited a significantly higher ergosterol content (15.9 ± 0.7 mg g-DCW^-1 ^v. 2.6 ± 0.07 mg g-DCW^-1^) as observed during glucose fermentation. For galactose cultivations, ergosterol content was only measured during transcriptome sampling, which occurred at 78 h for S288c (co-consumption of ethanol and galactose observed), and 35 h for CEN.PK113-7D. The total ergosterol content on galactose was 6.1 ± 0.04 mg g-DCW^-1 ^and 4.6 ± 0.2 mg g-DCW^-1 ^for CEN.PK113-7D and S288c, respectively.

### Transcriptome Characterization

Differential gene expression between S288c and CEN.PK113-7D, cultivated on both glucose and galactose, is summarized in Table 4. The GO characterization (process, function, component) for the comparative conditions S288c v. CEN.PK113-7D cultivated on glucose, and S288c v. CEN.PK113-7D cultivated on galactose, and divided into log_2_-fold change (lfc) > 0 and < 0, is presented in Additional file 1 Figure S7 and Figure S8. The metabolic pathway expression maps for each comparative condition are included in Additional file 1 Figure S9 and Figure S10. Lastly, all genes exhibiting statistically significant differential gene expression (*p_adj _*< 0.01) and having either a silent or nonsynonymous SNP are included in Additional file 1 Table S1 and Table S2.

**Table 4:** Summary of Differential Gene Expression

Summary of Differential Expression *(p_adj _*< 0.01)	S288c v. CEN.PK113-7D	
	*Glucose (*n *= 2)*	*Galactose (*n *= 2)*
Total No. of Differentially Expressed Genes	272	501
		
No. of Genes LFC > 0	204	337
LFC ± SD	2.13 ± 1.41	1.81 ± 1.17
No. of Genes with SNPs Detected	13	17
No. of Genes with Nonsynonymous SNPs	7	10
		
No. of Genes LFC < 0		
LFC ± SD	-2.12 ± 1.32	-1.53 ± 1.05
No. of Genes with SNPs Detected	4	4
No. of Genes with Nonsynonymous SNPs	1	0

**Notes: **Single nucleotide polymorphism (SNP). Standard deviation (SD). Nonsynonymous SNPs defined as a nucleotide modification results in a translated amino acid modification.

For the condition S288c v. CEN.PK113-7D cultivated on glucose, the top 272 differentially expressed genes, ranked according to *p_adj _*value are characterized into GO process terms largely dominated by responses to stimuli and pheromone, with the dominant metabolic process categories being trehalose metabolism, steroid metabolism, and amino acid transport. Specific genes consistent with this categorization high in *p_adj _*value rank and lfc > 0 are *GSY1 *(glycogen synthase, lfc 2.0, *p_adj _*value rank 23) and for lfc < 0 is *HMG1 *(HMG-CoA reductase, lfc -1.7, *p_adj _*value rank 14). For the condition S288c v. CEN.PK113-7D cultivated on galactose, the top 501 differentially expressed genes, ranked according to *p_adj _*value are characterized into GO process terms response to simuli and stress, carbohydrate metabolism, and transport. Specific metabolic genes noteworthy in this category, high in *p_adj _*value rank amongst genes with lfc > 0 include *MDH2 *(malate dehydrogenase, lfc 2.8, *p_adj _*value rank 8), *FBP1 *(fructose-1,6-bisphosphatase, lfc 4.2, *p_adj _*value rank 15), *GAD1 *(glutamate decarboxylase, lfc 3.0, *p_adj _*value rank 30), *GDH3*(NADP^+ ^dependent glutamate dehydrogenase, lfc 3.2, *p_adj _*value rank 32)*, GSY1 *(lfc 1.4, *p_adj _*value rank 41), and *ICL1 *(isocitrate lyase, lfc 2.7, *p_adj _*value rank 54). Similarly, specific metabolic genes high in *p_adj _*value rank amongst genes with lfc < 0 include *ARE2 *(acyl-coA:sterol acetyltransferase, lfc -2.3, *p_adj _*value rank 10), and *CYB5 *(cytochrome b5, lfc -1.6, *p_adj _*value rank 47).

In an effort to further investigate if larger regulatory mechanisms could be identified the list of genes exhibiting significant differential expression were submitted to the Yeast Search for Transcriptional Regulators And Consensus Tracking (YEASTRACT) curated repository of associations between transcription factors and target genes in *S. cerevisiae *[25,26]. The transcription factor, Tec1p, was identified as directly regulating 21.1% of the total submitted gene list (See Table 4, 272 genes, S288c glucose v. CEN.PK113-7D glucose), and was 1.7-fold higher expressed in CEN.PK113-7D compared to S288c (*p_adj _*value = 7.2 × 10^-3^). Tec1p was the only identified transcription factor to be significantly differentially expressed, and strongly regulates *FLO11*, a flocculin gene required for invasive growth, and pseudohyphal formation [27]. The transcription factors regulating the highest percentage of the differentially expressed genes, yet not being differentially expressed themselves, were Sok2p and Ste12p, with 32.5% and 21.5%, respectively, of submitted genes being directly regulated. Sok2p and Ste12p are transcription factors negatively regulating pseudohyphal differentiation [10]. A similar analysis was performed for galactose; however, similar results were obtained, with Sok2p and Ste12p directly regulating 23.1% and 17.4%, respectively, of the 501 differentially expressed genes (See Table 4). The transcription factors differentially expressed themselves were Msa1p (*p_adj _*value = 1.37 × 10^-1^) and Msa2p (*p_adj _*value = 1.65 × 10^-1^), putative G1-specific cell cycle transcription activators, and Usv1p, a putative zinc finger transcription factor regulating growth on non-fermentable carbon sources. *USV1 *expression was 2.2-fold higher in CEN.PK113-7D compared to S288c (*p_adj _*value = 3.6 × 10^-3^). Although relatively little is known about Usv1p, it has been shown to be induced post-diauxic shift, consistent with the deficiency in post-diauxic shift metabolism observed in S288c [28]. With the exception of Usv1p, all transcription factors identified are more closely related to the significant difference in growth rates between strains rather than their respiro-fermentative metabolism. Metabolic SNPs identified and subsequent analysis did not identify clear correlations or pathway enrichment that could explain the lack of respiro-fermentative metabolism in S288c. Metabolic genes containing nonsynonymous SNPs in CEN.PK113-7D, significantly differentially expressed on galactose, and related to oxidative metabolism included *ACS1 *(Acetyl-CoA synthetase), *GAD1 *(Glutamate decarboxylase), *YAT2 *(Carnitine acetyltransferase), and *CCP1 *(Mitochondrial cytochrome-c peroxidase) which were 7.8-fold, 3.2-fold, 4.5-fold, and 2.1-fold higher in CEN.PK113-7D, respectively (See Additional file 1 Table S2).

## Discussion

The physiological characterization clearly suggests that S288c has a deficiency in metabolism of respiro-fermentative carbon sources, such as ethanol and galactose, when compared to CEN.PK113-7D. Inspection of the significantly differentially expressed genes between strains cultivated on glucose or galactose did not reveal an obvious gene cluster that would explain this significant physiological difference. This is supported both by the GO characterization and pathway expression mapping.

There were two central carbon metabolic pathways enriched with nonsynonymous SNPs that also correlated with significant differences in phenotype. *S. cerevisiae *CEN.PK113-7D exhibited significantly higher ergosterol content during growth on glucose, and to a lesser extent, galactose. This is consistent with previous work where CEN.PK2-1C had very high ergosterol/erg-ester (20.0 mg g-DCW^-1^) and triacylglycerols content (15.2 mg g-DCW^-1^) compared to 9 other *S. cerevisiae *strains, including FY169 (ergosterol/erg-ester content: 8.5 mg g-DCW^-1^; triacylglycerols content: 2.4 mg g-DCW^-1^) which is isogenic to S288c [29,30]. The ergosterol biosynthetic pathway had significant nonsynonymous SNPs identified in *ERG8 *and *ERG9*, and silent SNPs identified in *ERG20 *and *HMG1*. Both *ERG8 *and *ERG9 *were not significantly differentially expressed, either in glucose or galactose, suggestive again that phenotypic observations, consistent with genome sequence variations, are not necessarily directly manifested at the transcriptome level. Both *ERG8 *(encodes phosophomevalonate kinase) and *ERG9 *(encodes squalene synthesase) are essential cytosolic enzymes in the biosynthetic pathway of isoprenoids and sterols (*Δerg8 *and *Δerg9*, were found to both be auxtrophic for ergosterol in the systematic deletion library), including ergosterol, from mevalonate [11,31,32]. The ergosterol biosynthetic pathway is highly regulated through feedback inhibition mechanisms and by several rate-controlling steps, including that catalyzed by HMG-CoA reductase, encoded by *HMG1 *[33,34]. Under both glucose and galactose, *HMG1 *expression was significantly down-regulated in S288c compared to CEN.PK113-7D by 3.2-fold (*p_adj _*value = 3.3 × 10^-4^) and 1.8-fold (*p_adj _*value = 8.6 × 10^-3^), respectively, correlating with the significantly less ergosterol content in S288c cultivated on glucose and to a lesser extent, on galactose. Furthermore, *ERG9 *has been previously identified as also having a regulatory role [35], consistent with the hypothesis that a nonsynonymous SNP resulting in altered protein function could affect ergosterol synthesis. *ERG8 *on the other hand has not been explicitly shown to have a regulatory function, yet, when the specific activity of 0.06 μmol min^-1 ^mg^-1 ^is compared to other ergosterol synthetic enzymes such as *ERG13 *(2.1 in *S. cerevisiae*), *ERG12 *(0.77 in *S. cerevisiae*), *ERG20 *(5.22 in *S. cerevisiae*), and especially the known regulator *HMG1/HMG2 *(0.0035 in *S. cerevisiae*), it is suggestive that *ERG8 *is likely a rate limiting step [36-44]. There were a large number of nonsynonymous SNPs that encoded significant changes in amino acid classes, further suggestive that *ERG8 *is a strong metabolic engineering target for understanding the significantly higher ergosterol content in CEN.PK113-7D. Lastly, the observation that neither *ERG8 *nor *ERG9 *were differentially expressed under glucose or galactose, suggests their potential affect on phenotype is likely post-translational.

Similar to ergosterol biosynthesis, the galactose uptake pathway phenotype in S288c was vastly lower rate compared to CEN.PK113-7D, correlating with the nonsynonymous SNP enrichment in *GAL1 *and *GAL10*, and silent SNPs in *GAL7*. Neither *GAL1 *(encodes galactokinase) nor *GAL10 *(encodes UDP-glucose-4-epimerase) were significantly differentially expressed during growth on galactose; however, on glucose *GAL1 *was significantly up-regulated (*p_adj _*value = 9.7 × 10^-4^), 2.9-fold in CEN.PK113-7D. Both *Δgal1 *and *Δgal10 *mutants are unable to grow on galactose as sole carbon sources [45-47]. The significant number of nonsynonymous SNPs in both essential galactose genes suggests obvious targets for explanation of why S288c is incapable of galactose respiro-fermentative metabolism. Furthermore, it should be noted that S288c has been described as *Δgal2 *(See Additional file 1 Table S3), which may be ascribed to the presence of 4 SNPs between CEN.PK113-7D and S288c in the coding region, and 14 SNPs in the upstream region that result in a poor functional Gal2p. However, clearly, S288c is able to co-metabolize galactose with ethanol and this may be ascribed by galactose transport by e.g. the hexose transporters.

A further metabolic engineering benefit of whole genome sequencing was the detection of a nonsynonymous SNP resulting in a stop codon of *PAD1 *(encodes phenylacrylic acid decarboxylase). Pad1p is essential for decarboxylation of aromatic carboxylic acids conferring resistance to cinnamic acid, and a nonsynonymous SNP was detected at nucleotide position 294 (T to G), resulting in a stop codon (TAT → TAG) [48]. Although Pad1 relevant phenotypes were not explored, the transcriptome response on glucose revealed significant differential expression of *PAD1 *(*p_adj _*value = 1.5 × 10^-3^), with 3.1-fold higher expression in S288c compared to CEN.PK113-7D. This is consistent with the stop codon detected in CEN.PK113-7D at position 294, noting that the total ORF genomic DNA sequence is 729 nucleotides, and therefore unlikely to be transcribed and detected.

## Conclusions

Whole high-throughput genome sequencing of *S. cerevisiae *S288c and CEN.PK113-7D resulted in identification of 13,787 filtered SNPs in CEN.PK113-7D, with a total of 939 SNPs detected across 158 unique metabolic genes, 85 of which contained a total of 219 nonsynonymous SNPs. There were two central carbon metabolic pathways enriched with nonsynonymous SNPs that also correlated with significant differences in phenotype. *S. cerevisiae *CEN.PK113-7D exhibited significantly higher ergosterol content during growth on glucose, and to a lesser extent, galactose. The ergosterol biosynthetic pathway had significant nonsynonymous SNPs identified in *ERG8 *and *ERG9*, and silent SNPs identified in *ERG20 *and *HMG1*. The flux through the galactose uptake pathway was much lower in S288c compared with CEN.PK113-7D, correlating with the nonsynonymous SNP enrichment in *GAL1 *and *GAL10*, and silent SNPs in *GAL7*. More globally, the physiological characterization clearly suggests that S288c has a deficiency in metabolism of respiratory carbon sources, such as ethanol and galactose, when compared to CEN.PK113-7D. Inspection of the significantly differentially expressed genes between strains cultivated on glucose or galactose did not reveal an obvious gene cluster that would explain this significant physiological difference. In summary and perhaps not surprisingly, transcriptome analysis did not provide a clear hypothesis for major phenotypes observed, suggesting that genotype to phenotype correlations are manifested post-transcriptionally or post-translationally either through protein concentration and/or function. Clearly, future work must validate these correlations through genetic engineering of identified SNPs in either S288c or CEN.PK113-7D to see if desired phenotypes, such as increased galactose uptake or ergosterol synthesis in S288c, are observed. Future work must also expand on the metabolic SNP analysis presented to include all 13,787 SNPs, realizing phenotypic observations may not necessarily be linked directly to metabolic SNPs, but rather SNPs affecting larger regulatory mechanisms and networks, such as those governed by transcription factors. Certainly, as *S. cerevisiae *continues to be exploited, particularly for metabolic engineering applications, the integration of physiological characterization, transcriptome analysis, and metabolic SNP detection with high-throughput whole genome sequencing provides direct correlations between observed phenotypes and genotypes and offers high probability of success metabolic targets.

## Methods

### Strain Description

*S. cerevisiae *strain S288c (American Type Culture Collection, ATCC^®^) and strain CEN.PK113-7D (Scientific Research and Development, SRD GmbH were used in this study. Genotype of strain S288c was described by Mortimer *et al*, [49], Johnston *et al *[50], Goffeau *et al *[10], Cherry *et al *[11] and genotype of strain CEN.PK113-7D was described by Cherry *et al *[11], van Dijken *et al *[24]. More information of *S. cerevisiae *strains was described in Additional file 1 Table S3.

### Genome Sequencing, Annotation and Analysis

#### DNA Isolation

A standard 500 mL shake flask, supplemented with 10 g L^-1 ^glucose and inoculated with a single colony of *S. cerevisiae *S288c or CEN.PK113-7D, was permitted to grow for 24-48 h at 30°C until visual inspection confirmed a high optical density. A total of 5 mL culture was aliquoted into 15 mL sterile tubes (one per extraction), centrifuged (4000 RCF) for 5 min, washed with 2 mL deionized water, and pelleted. Cell pellets were resuspended in 0.5 mL lysis buffer. Lysis buffer consisted of 0.1 M Tris pH 8.0, 50 mM EDTA, and 1% SDS final concentration. The lysis buffer suspension was transferred to a 1.5 mL FastPrep screw cap tube, to which 200 μL acid-washed glass beads (250-500 μm) and 25 μL 5 M NaCl was added. A FastPrep™ FP120 (QBiogene, Irwine, CA) was used for cell lysis, with two cycles of 20 s disruption and 1 min on ice. The resulting cell suspension was centrifuged (13,000 RCF) for 10 min., and the resulting clear liquid, approximately 350 μL, avoiding white cell debris and beads, was aspirated with a pipette and transferred to 1.5 mL microcentrifuge tubes. 400 μL chloroform (TE-saturated) was added to each tube, mixed, and a chloroform extraction performed. 1 mL 99% ethanol was added to the resulting suspension, mixed, centrifuged (13,000 RCF) for 6 min., ethanol decanted, and then resuspended in 70% ethanol. The resulting suspension was centrifuged (13,000 RCF) for 6 min., ethanol decanted, and pellet permitted to dry for 25-60 min. The pellet was then resuspended in 50 μL 2 mM Tris, incubated for 10 min. at 37°C, and stored at -20°C.

#### Illumina/Solexa Genome Sequencing and SNP Analysis

Isolated DNA from *S. cerevisiae *S288c and CEN.PK113-7D were processed to prepare short insert shotgun libraries at Fasteris SA (Geneva, Switzerland), utilizing the Solexa technology according to the manufacturer's recommendations (Illumina). The whole-genome sequencing was performed in 2007 on a Genome Analyzer "classic" instrument with sequencing kits version 1 and base calling on the Solexa Pipeline (version 0.2.2.5). SNP detection was performed using two independent approaches: mapping on the S288c reference sequence and *de novo *assembly. The MAQ software package http://maq.sourceforge.net was used to map the short base paired-reads (35 bp) to the reference genome *S. cerevisiae *S288c version 12.0, available at the *Saccharomyces *Genome Database (SGD). For consensus calling, the *maq assemble -m *command was used to call the consensus sequences from read mapping. The value *m*set to 1 specifies the maximum numbers of mismatches allowed for a filtered read. To detect high-quality SNP identification, the command *maq.pl SNPfilter *was set up. The threshold values applied to SNP detection for the CEN.PK113-7D sequence relative to the reference sequence were read depth (*-d*) > 5 for few reads, read depth (*-D*) < 255 for too many reads, uniqueness of the region (-*w*) < 1.5, quality of the read (*-Q*) > 50, and best read quality (-*n*) > 40. These threshold parameter values were tested such that the amount of coverage and proportion of genome with aligned sequences was maximized, and a graphical representation of SNPs was produced to confirm results. The *de novo *assembly has been performed using the EDENA software package http://www.genomic.ch/edena.php as described previously [21]. The EDENA assembly results for both whole genome sequencing and for SNP detection in metabolic genes are presented in Additional file 1, Table S4. The *de novo *assembly used for SNP detection was essentially used for an independent verification of the SNPs detected by mapping on the reference sequence, as not all the genome was represented. Indeed a higher coverage, as well as longer insert libraries are required to achieve more complete *de novo *assembly. For purposes of the subsequent SNP analysis only the MAQ software results were used. The FASTA files of each genome sequence are available upon request.

All metabolic genes containing SNPs, both silent and nonsynonymous, were manipulated within the software BioEdit v7.08 http://www.mbio.ncsu.edu/BioEdit/bioedit.html. Specifically, the ORF genomics nucleotide sequence available on *Saccharomyces *Genome Database (SGD) http://www.yeastgenome.org were imported into BioEdit, and the sequences modified with the identified SNP, creating a new CEN.PK113-7D sequence for that ORF relative to the original S288c strain. Both the S288c and CEN.PK113-7D nucleotide sequences were then translated in fix full frames, and amino acid polymorphisms were identified, leading to the categorization of each SNP as either being silent or nonsynonymous. Subsequent physiological characterization of the gene and all relevant amino acid information from UnitProt were managed in a spreadsheet using Microsoft Excel. Multi-sequence Pfam alignments were performed using a custom BioPerl script and the UNIX operating environment. Calculations and characterization described in Additional file 1, Figure S3, related to amino acids, were then performed using Microsoft Excel.

#### Gene Prediction and Functional Annotation

Genes were predicted in the *S. cerevisiae *strain CEN.PK113-7D genome based on the homologies to known or putative genes in the public database of the *S. cerevisiae *S288c v12.0, sequence available at the *Saccharomyces *Genome Database (SGD). The statistical features of the genes were predicted by a combination of gene-finding software. To perform annotation, BLASTN and GlimmerHMM were applied. GlimmerHMM was used for prediction the gene structures of strain CEN.PK113-7D by training the gene models from the strain S288c. All of the predicted protein-coding genes were annotated by searching against the SGD database using BLASTP, followed by manual curation.

#### Development of Genome Browser and SNPs Viewer

The genome browser and SNP viewer were implemented by PHP source code and MySQL database management in our hosting server http://members.dot5hosting.com/controlpanel/. Currently, our MySQL database structure contains different features that correspond to chromosome location, gene position, gene function, GO process, metabolism, and SNPs.

### Fermentation

#### Medium Formulation

A chemically defined minimal medium of composition 5.0 g L^-1 ^(NH_4_)_2_SO_4_, 3.0 g L^-1 ^KH_2_PO_4_, 0.5 g L^-1 ^MgSO_4_.7H_2_O, 1.0 mL L^-1 ^trace metal solution, 300 mg L^-1 ^uracil, 0.05 g L^-1 ^antifoam 204 (Sigma-Aldrich A-8311), and 1.0 mL L^-1 ^vitamin solution was used for all shake flask and 2L well-controlled fermentations [51]. The trace elment solution included 15 g L^-1 ^EDTA, 0.45 g L^-1 ^CaCl_2_.2H_2_O, 0.45 g L^-1 ^ZnSO_4 _.7H_2_O, 0.3 g L^-1 ^FeSO_4_.7H_2_O, 100 mg L^-1 ^H_3_BO_4_, 1 g L^-1 ^MnCl_2_.2H_2_O, 0.3 g L^-1 ^CoCl_2_.6H_2_O, 0.3 g L^-1 ^CuSO_4_.5H_2_O, 0.4 g L^-1 ^NaMoO_4_.2H_2_O. The pH of the trace metal solution was adjusted to 4.0 with 2 M NaOH and heat sterilized. The vitamin solution included 50 mg L^-1 ^d-biotin, 200 mg L^-1 ^*para-*amino benzoic acid, 1 g L^-1 ^nicotinic acid, 1 g L^-1 ^Ca.pantothenate, 1 g L^-1 ^pyridoxine HCl, 1 g L^-1 ^thiamine HCl, and 25 mg L^-1 ^m.inositol. The pH of the vitamin solution was adjusted to 6.5 with 2 M NaOH, sterile-filtered and the solution was stored at 4°C. The final formulated medium, excluding glucose and vitamin solution supplementation, is adjusted to pH 5.0 with 2 M NaOH and heat sterilized. For carbon-limited cultivations the sterilized medium is supplemented with 40 g L^-1 ^glucose or 40 g L^-1 ^galactose, heat sterilized separately, and 1.0 mL L^-1 ^vitamin solution is added by sterile filtration (0.20 μm pore size Ministart^®^-Plus Sartorius AG, Goettingen, Germany).

#### Shake Flask Cultivations and Stirred Tank Fermentations

Shake flask cultivations were completed in 500 mL Erlenmeyer flasks with two diametrically opposed baffles and two side-necks with septums for sampling by syringe. Flasks were heat sterilized with 100 mL of medium, inoculated with a single colony, and incubated at 30°C with orbital shaking at 150 RPM. Stirred tank fermentations were completed in well-controlled, aerobic, 2.2L Braun Biotech Biostat B fermentation systems with a working volume of 2L (Sartorius AG, Goettingen, Germany). The temperature was controlled at 30°C. The fermenters were outfitted with two disk-turbine impellers rotating at 600 RPM. Dissolved oxygen was monitored with an autoclavable polarographic oxygen electrode (Mettler-Toledo, Columbus, OH). During aerobic cultivation the air sparging flow rate was 2 vvm. The pH was kept constant at 5.0 by automatic addition of 2 M KOH. Off-gas passed through a condenser to minimize the evaporation from the fermenter. The fermenters were inoculated from shake flask precultures to an initial OD_600 _0.01. Two biological replicates were performed for each fermentation condition.

#### Off-gas Analysis

The effluent fermentation gas was measured every 30 seconds for determination of O_2_(g) and CO_2_(g) concentrations by the off-gas analyzer Brüel and Kjær 1308 (Brüel & Kjær, Nærum, Denmark).

#### Biomass Determination

The optical density (OD) was determined at 600 nm using a Shimadzu UV mini 1240 spectrophotometer (Shidmazu Europe GmbH, Duisberg, Germany). Duplicate samples were diluted with deionized water to obtain OD_600 _measurements in the linear range of 0-0.4 OD_600 _Samples were always maintained at 4°C post-sampling until OD_600 _and dry cell weight (DCW) measurements were performed. DCW measurements were determined through the exponential phase, until stationary phase was confirmed according to OD_600 _and off-gas analysis. Nitrocellulose filters (0.45 μm Sartorius AG, Goettingen, Germany) were used. The filters were pre-dried in a microwave oven at 150W for 10 min., and cooled in a dessicator for 10 min. 5.0 mL of fermentation broth were filtered, followed by 10 mL DI water. Filters were then dried in a microwave oven for 20 min. at 150W, cooled for 15 min. in a desiccator, and the mass was determined.

#### Metabolite Concentration Determination

All fermentation samples were immediately filtered using a 0.45 μm syringe-filter (Sartorius AG, Goettingen, Germany) and stored at -20°C until further analysis. Glucose, ethanol, glycerol, acetate, succinate, pyruvate, fumarate, citrate, oxalate, and malate were determined by HPLC analysis using an Aminex HPX-87 H ion-exclusion column (Bio-Rad Laboratories, Hercules, CA). The column was maintained at 65°C and elution performed using 5 mM H_2_SO_4 _as the mobile phase at a flow rate of 0.6 mL min^-1^. Glucose, ethanol, glycerol, acetate, succinate, citrate, fumarate, malate, oxalate were detected on a Waters 410 differential refractometer detector (Shodex, Kawasaki, Japan), and acetate and pyruvate were detected on a Waters 468 absorbance detector set at 210 nm. Ergosterol measurements were made according to previous published methods [52].

### Transcriptomics

#### RNA Sampling and Isolation

Samples for RNA isolation from two biological replicates from the late-exponential phase of glucose-limited and galactose-limited batch cultivations were taken by rapidly sampling 25 mL of culture into a 50 mL sterile Falcon tube with 40 mL of crushed ice in order to decrease the sample temperature to below 2°C in less than 10 s. Cells were immediately centrifuged (4000 RCF at 0°C for 2.5 min.), the supernatant discarded, and the pellet frozen in liquid nitrogen and it was stored at -80°C until total RNA extraction. Total RNA was extracted using the FastRNA Pro RED kit (QBiogene, Carlsbad, USA) according to manufacturer's instructions after partially thawing the samples on ice. RNA sample integrity and quality was determined prior to hybridization with an Agilent 2100 Bioanalyzer and RNA 6000 Nano LabChip kit according to the manufacturer's instruction (Agilent, Santa Clara, CA).

#### Probe Preparation and Hybridization to DNA Microarrays

mRNA extraction, cDNA synthesis, labeling, and array hybridization to Affymetrix Yeast Genome Y2.0 arrays were performed according to the manufacturer's recommendations (Affymetrix GeneChip^® ^Expression Analysis Technical Manual, 2005-2006 Revision 2.0). Washing and staining of arrays were performed using the GeneChip Fluidics Station 450 and scanning with the Affymetrix GeneArray Scanner (Affymetrix, Santa Clara, CA).

#### Microarray Gene Transcription Analysis

Affymetrix Microarray Suite v5.0 was used to generate CEL files of the scanned DNA microarrays. These CEL files were then processed using the statistical language and environment R v5.3 (R Development Core Team, 2007, http://www.r-project.org), supplemented with Bioconductor v2.3 (Biconductor Development Core Team, 2008, http://www.bioconductor.org) packages Biobase, affy, gcrma, and limma [53]. The probe intensities were normalized for background using the robust multiarray average (RMA) method only using perfect match (PM) probes after the raw image file of the DNA microarray was visually inspected for acceptable quality. Normalization was performed using the qspline method and gene expression values were calculated from PM probes with the median polish summary. Statistical analysis was applied to determine differentially expressed genes using the limma statistical package. Moderated *t-*tests between the sets of experiments were used for pair-wise comparisons. Empirical Bayesian statistics were used to moderate the standard errors within each gene and Benjamini-Hochberg's method was used to adjust for multi-testing. A cut-off value of adjusted *p *< 0.01 was used for statistical significance [54]. Statistically significant differential gene expression lists were then submitted to the GO Term Finder (version 0.83) of the *Saccharomyces *Genome Database (SGD) for GO process, function, and component statistically significant identification (*p *< 0.01). Furthermore, the same differential gene expression lists were submitted to the Expression Viewer (Pathway Tools version 12.0 generated by SRI International on SGD) for metabolic pathway mapping and identification [11].

#### Data Deposition

Genome sequence and annotated gene of *S. cerevisiae *CEN.PK113-7D were deposited at sysbio database http://www.sysbio.se/cenpk. Normalized gene expression data were deposited at the GEO database http://www.ncbi.nlm.nih.gov/geo/ with accession number GPL2529 (platform), GSM536874-GSM536881 (samples), and GSE21479 (series).

## Authors' contributions

JMO, WV, MAA, RO, JM, LF, LB, MØ, MS, AC, and JN all contributed to the conception and design of the study. JMO and MAA performed all fermentation and physiological characterization. JMO performed transcriptome measurements and analysis. LF, LB, and MØ performed high-throughput genome sequencing. JMO, WV, MAA, RO, and JM performed genome and phenotype annotation. WV developed the genome browser and other bioinformatics tools. JMO, WV, MAA, RO, JM, LF, MS, AC, and JN all contributed to the overall study analysis, drafting, and reviewing of the manuscript. All authors read and approved the final manuscript.

## Supplementary Material

Additional file 1 **Additional Files**. Figure S1. Title: Gene Ontology terms for SNP characterization. Description: Gene Ontology (GO) function and component terms for the nonsynonymous SNPs identified in CEN.PK113-7D compared to S288c. Figure S2. Title: SNP enrichment in *S. cerevisiae *metabolism. Description: The metabolic map, produced using the *Saccharomyces *Genome Database (SGD) Expression Viewer (SRI International Pathway Tools version 12.0, based upon *S. cerevisiae *S288c, version 12.0) was created using the SNP data produced for CEN.PK113-7D compared to S288c. Figure S3. Title: Methodology for SNP characterization at amino acid level. Description: The flow-diagram describes the bioinformatics approach taken to estimate the likelihood of occurrence of a nonsynonymous SNP in CEN.PK113-7D or S288c. Figure S4. Title: Conservation distance. Description: The *Conservation Distance*, previously described in Additional file 1, Figure S3, is plotted for all 210 nonsynonymous SNPs. Figure S5. Title: SNP analysis of ERG8 at nucleotide positions 49 and 247. Description: The gene *ERG8 *of the ergosterol synthesis pathway contains a total of 4 nonsynonymous SNPs, two of which, located at nucleotide positions 49 and 247, are analyzed. Figure S6. Title: Amino acid characterization of SNPs detected between S288c and CEN.PK113-7D. Description: Amino Acid Characterization-CEN.PK113-7D vs S288c Profiles. Figure S7. Title: Gene Ontology terms for S288c v. CEN.PK113-7D (Glucose). Description: Gene Ontology (GO) process, function, and component terms for differentially expressed genes of S288c vs. CEN.PK113-7D cultivated on glucose. Figure S8. Title: Gene Ontology terms for S288c vs. CEN.PK113-7D (Galactose). Description: Gene ontology (GO) process, function, and component terms for differentially expressed genes of S288c vs. CEN.PK113-7D cultivated on galactose. Figure S9. Title: Metabolic pathway expression mapping for S288c Glucose vs. CEN.PK113-7D Glucose. Description: The metabolic map produced using the *Saccharomyces *Genome Database (SGD) Expression Viewer (SRI International Pathway Tools version 12.0, based upon *S. cerevisiae *S288c, version 12.0) was created using statistically significant log_2_-fold expression values for S288c glucose vs. CEN.PK113-7D glucose. Figure S10. Title: Metabolic pathway expression mapping for S288c Galactose vs. CEN.PK113-7D Galactose. Description: The metabolic map produced using the *Saccharomyces *Genome Database (SGD) Expression Viewer (SRI International Pathway Tools version 12.0, based upon *S. cerevisiae *S288c, version 12.0) was created using statistically significant log_2_-fold expression values for S288c galactose vs. CEN.PK113-7D galactose. Table S1. Title: Transcriptome-S288c v CEN.PK113-7D Glucose. Description: List of significant genes (*p_adj_*< 0.01). Table S2. Title: Transcriptome-S288c v CEN.PK113-7D Galactose. Description: List of significant genes (*p_adj _*< 0.01). Table S3. Title: Description of *S. cerevisiae *strains. Table S4. Title: EDENA determination for *de novo *assembly of S288c and CEN.PK113-7D sequences Click here for file
