# Supplementary material for: Whole genome sequencing of Saccharomyces cerevisiae: from genotype to phenotype for improved metabolic engineering applications
Source: BMC Genomics. 2010 Dec 22;11:723. doi: 10.1186/1471-2164-11-723 (PMC3022925; doi:10.1186/1471-2164-11-723)
Supplement: Additional file 1 — Additional Files. Figure S1. Title: Gene Ontology terms for SNP characterization. Description: Gene Ontology (GO) function and component terms for the nonsynonymous SNPs identified in CEN.PK113-7D compared to S288c. Figure S2. Title: SNP enrichment in S. cerevisiae metabolism. Description: The metabolic map, produced using the Saccharomyces Genome Database (SGD) Expression Viewer (SRI International Pathway Tools version 12.0, based upon S. cerevisiae S288c, version 12.0) was created using the SNP data produced for CEN.PK113-7D compared to S288c. Figure S3. Title: Methodology for SNP characterization at amino acid level. Description: The flow-diagram describes the bioinformatics approach taken to estimate the likelihood of occurrence of a nonsynonymous SNP in CEN.PK113-7D or S288c. Figure S4. Title: Conservation distance. Description: The Conservation Distance, previously described in Additional file 1, Figure S3, is plotted for all 210 nonsynonymous SNPs. Figure S5. Title: SNP analysis of ERG8 at nucleotide positions 49 and 247. Description: The gene ERG8 of the ergosterol synthesis pathway contains a total of 4 nonsynonymous SNPs, two of which, located at nucleotide positions 49 and 247, are analyzed. Figure S6. Title: Amino acid characterization of SNPs detected between S288c and CEN.PK113-7D. Description: Amino Acid Characterization-CEN.PK113-7D vs S288c Profiles. Figure S7. Title: Gene Ontology terms for S288c v. CEN.PK113-7D (Glucose). Description: Gene Ontology (GO) process, function, and component terms for differentially expressed genes of S288c vs. CEN.PK113-7D cultivated on glucose. Figure S8. Title: Gene Ontology terms for S288c vs. CEN.PK113-7D (Galactose). Description: Gene ontology (GO) process, function, and component terms for differentially expressed genes of S288c vs. CEN.PK113-7D cultivated on galactose. Figure S9. Title: Metabolic pathway expression mapping for S288c Glucose vs. CEN.PK113-7D Glucose. Description: The metabolic map produced using [file 1471-2164-11-723-S1.DOC]

**Additional file 1**

**Whole genome sequencing of *Saccharomyces cerevisiae*: from genotype to phenotype for improved metabolic engineering applications**

José Manuel Otero^1,2,5,#^, Wanwipa Vongsangnak^1,2,8,#^, Mohammad A. Asadollahi^1,2,6^ , Roberto Olivares-Hernandes^1,2^, Jérôme Maury^2,7^, Laurent Farinelli^3^, Loïc Barlocher^3^, Magne Østerås^3^, Michel Schalk^4^, Anthony Clark^4^, Jens Nielsen^1,2,*^

^1^Department of Chemical and Biological Engineering, Chalmers University of Technology, SE-41296 Gothenburg, Sweden.

^2^Center for Microbial Biotechnology, Department of Systems Biology, Technical University of Denmark DK-2800, Kgs. Lyngby, Denmark.

^3^Fasteris SA, Geneva, Switzerland.

^4^Firmenich SA, Corporate Research & Development Division, Geneva, Switzerland.

^5^Vaccine & Biologics Process Development, Vaccine Research & Development, Merck Research Labs, West Point, PA, USA.

^6^Biotechnology Group, Faculty of Advanced Sciences and Technologies, University of Isfahan, Isfahan 81746-73441, Iran.

^7^Fluxome Sciencies A/S, Research & Development, DK-3660 Stenlose, Denmark.

^8^Current Address: Center for Systems Biology, Soochow University, Suzhou 215006, China.

^#^These authors contributed equally to this work

*Corresponding author

**Content**

1. **Figure S1:** Gene Ontology terms for SNP characterization.
2. **Figure S2:** SNP enrichment in *S. cerevisiae* metabolism.
3. **Figure S3:** Methodology for SNP characterization at amino acid level.
4. **Figure S4:** Conservation distance.
5. **Figure S5:** SNP analysis of ERG8 at nucleotide positions 49 and 247.
6. **Figure S6:** Amino acid characterization of SNPs detected between S288c and CEN.PK113-7D.
7. **Figure S7:** Gene ontology terms for S288c v. CEN.PK113-7D (Glucose).
8. **Figure S8:** Gene ontology terms for S288c vs. CEN.PK113-7D (Galactose).
9. **Figure S9:** Metabolic pathway expression mapping for S288c Glucose vs. CEN.PK113-7D Glucose.
10. **Figure S10:** Metabolic pathway expression mapping for S288c Galactose vs. CEN.PK113-7D Galactose.
11. **Table S1:** Transcriptome – S288c v CEN.PK113-7D Glucose.
12. **Table S2:** Transcriptome – S288c v CEN.PK113-7D Galactose.
13. **Table S3:** Description of *S. cerevisiae* strains
14. **Table S4:** EDENA determination for *de novo* assembly of S288c and CEN.PK113-7D sequences.

**GO Function, Nonsynonymous SNP characterization**

**GO Component, Nonsynonymous SNP characterization**

**Figure S1.**  Gene Ontology (GO) function and component terms for the nonsynonymous SNPs identified in CEN.PK113-7D compared to S288c. The *x­*-axis in log-scale displays both the significance of each category (*p* < 0.01, symbol: solid black), and the number of genes from the total of 85 containing nonsynonymous SNPs (symbol: solid white). GO process characterization performed using the *Saccharomyces* Genome Database (SGD).

**Figure S2.** SNP enrichment in *S. cerevisiae* metabolism.

The metabolic map, produced using the *Saccharomyces* Genome Database (SGD) Expression Viewer (SRI International Pathway Tools version 12.0, based upon *S. cerevisiae* S288c, version 12.0) was created using the SNP data produced for CEN.PK113-7D compared to S288c. Pathways in red indicate nonsynonymous SNPs (85 genes) while those in blue indicate silent SNPs (73 genes). Note that number of genes does not necessarily coincide with number of pathways due to iso-enzymes.

**Figure S3.** The above flow-diagram describes the bioinformatics approach taken to estimate the likelihood of occurrence of a nonsynonymous SNP in CEN.PK113-7D or S288c. Specifically, the top 10 homologous protein sequences based on Pfam search and multiple alignments were determined. Each amino acid the SNP position identified was catalogued and characterized. Specifically, indices referred to as the *S288c Frequency*, *CEN.PK Frequency*, and *Conservation Distance* was calculated. The *Conservation Distance*, bound from -1 to 1, is a convenient measure of whether the nucleotide detected in CEN.PK113-7D or S288c is dominant compared to homologous sequences. Resulting amino acids were then characterized according to their physical chemistry properties.


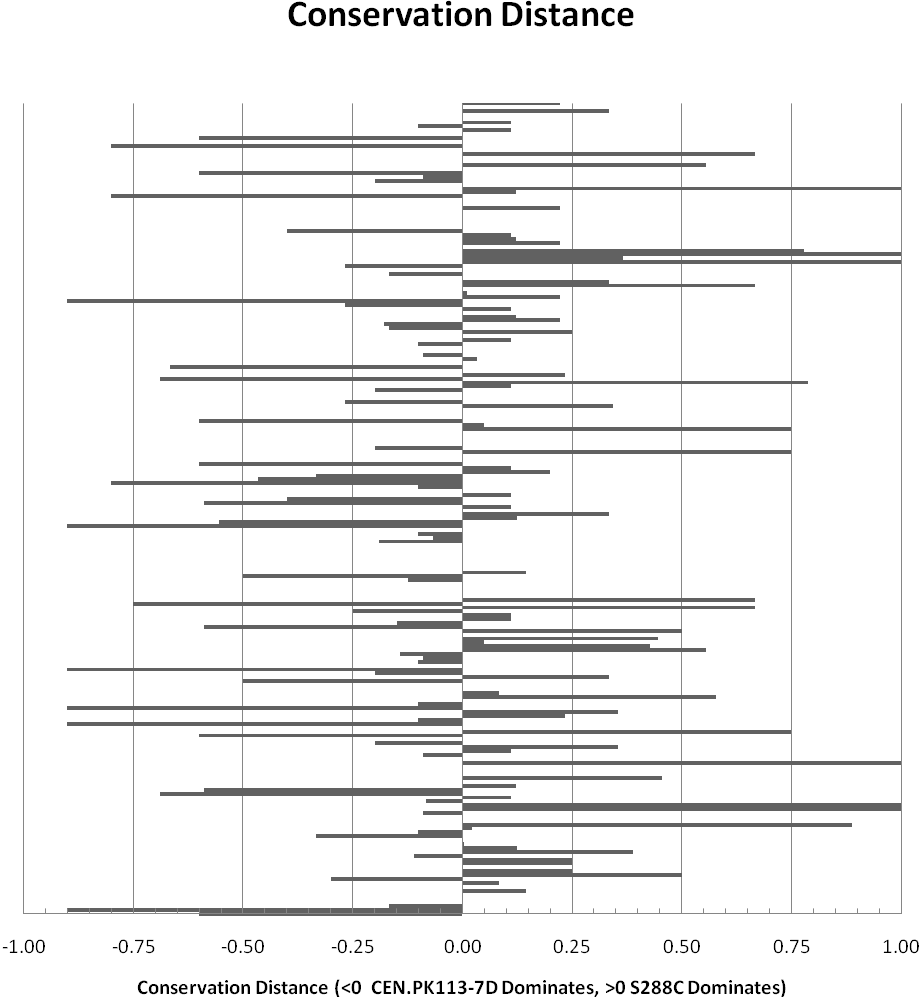


**Conservation Distance Average ± SD = 0.03 ± 0.40**

**Figure S4.**  The Conservation Distance, previously described in Additional file 1, Figure S3, is plotted for all 210 nonsynonymous SNPs.

**Figure S5.** The gene *ERG8* of the ergosterol synthesis pathway contains a total of 4 nonsynonymous SNPs, two of which, located at nucleotide positions 49 and 247, are analyzed here. The top plots show the CEN.PK Match Frequency, Dominant AA Frequency, S288c Match Frequency, and Conversation Distance. The middle plots show the frequency (fraction) of each categorization across the amino acid sequences resulting from Pfam multi-sequence alignment. The bottom plots show the characterization of the original S288c amino acid (symbol: blue bar) and the CEN.PK113-7D amino acid (symbol: red bar).

**Figure S6.** Brief Description of Amino Acid Characterization – CEN.PK113-7D vs S288c Profiles

In the figure that follows, the same amino acid characterization as presented earlier was performed for only the CEN.PK113-7D sequence (referred to as “SNP”), and the S288c sequence (referred to as “SGD”). The scoring system was a simple binary assignment, where 0 indicated the amino acid did not fall into the category or 1 indicating that it did. Therefore, the plots that follow should have the following interpretation:

- *If the value of the individual amino acid is zero, than neither the SNP (CEN.PK113-7D) or SGD (S288c) amino acid qualify for that category*
- *If the value of the individual amino acid is 1, then only one of the sequences – SNP (CEN.PK113-7D) or SGD (S288c) – fall into the category, suggesting a change in characterization. The color codes should then be used to determine which sequence falls into the category.*
- *If the value of the individual amino acid is 2, then both sequences fall into that functional category.*

Note that on the y-axis is the individual nonsynonymous SNPs. The naming nomenclature used is “GeneSNP”, such that for the example “BET2143”, the gene is *BET2* and the SNP is at nucleotide position 143. Due to the large number of SNPs each individual name is not included. Therefore, the plots that follow intend to provide a visual perspective for how many nonsynonymous SNPs resulted in a functional amino acid change.

**Figure S7.** Gene Ontology (GO) process, function, and component terms for differentially expressed genes of S288c vs. CEN.PK113-7D cultivated on glucose. The *x­*-axis in log-scale displays both the significance of each category (*p* < 0.01, symbol: solid black), and the number of differentially expressed genes in each GO category (symbol: solid white). The GO terms were determined separately for genes expressing positive and negative log_2_-fold change (LFC). GO characterization performed using the *Saccharomyces* Genome Database (SGD).

**Figure S8.** Gene ontology (GO) process, function, and component terms for differentially expressed genes of S288c vs. CEN.PK113-7D cultivated on galactose. The *x­*-axis in log-scale displays both the significance of each category (*p* < 0.01, symbol: solid black), and the number of differentially expressed genes in each GO category (symbol: solid white). The GO terms were determined separately for genes expressing positive and negative log-fold change (LFC). GO characterization performed using the *Saccharomyces* Genome Database (SGD).

Figure S9. The metabolic map produced using the *Saccharomyces* Genome Database (SGD) Expression Viewer (SRI International Pathway Tools version 12.0, based upon *S. cerevisiae* S288c, version 12.0) was created using statistically significant log_2_-fold expression values for S288c glucose vs. CEN.PK113-7D glucose.

**Figure S10.** The metabolic map produced using the *Saccharomyces* Genome Database (SGD) Expression Viewer (SRI International Pathway Tools version 12.0, based upon *S. cerevisiae* S288c, version 12.0) was created using statistically significant log_2_-fold expression values for S288c galactose vs. CEN.PK113-7D galactose.

| **Table S1.** Transcriptome: S288c v CEN.PK113-7D Glucose (*p_adj_*< 0.01) | | | | | | |  | |  |
| --- | --- | --- | --- | --- | --- | --- | --- | --- | --- |
| **Systematic Gene Name** | **logFC** | **p_adj_ value** | **Standard Gene Name** | **Description^1^** | **Silent SNP** | **Nonsynonymous SNP** | |  | |
| YBR001C | 1.67 | 3.55E-04 | *NTH2* | Putative neutral trehalase, required for thermotolerance and may mediate resistance to other cellular stresses |  | Y | |  | |
| YMR250W | 2.04 | 7.12E-04 | *GAD1* | Glutamate decarboxylase, converts glutamate into gamma-aminobutyric acid (GABA) during glutamate catabolism; involved in response to oxidative stress |  | Y | |  | |
| YML100W | 2.61 | 7.41E-04 | *TSL1* | Large subunit of trehalose 6-phosphate synthase (Tps1p)/phosphatase (Tps2p) complex, which converts uridine-5'-diphosphoglucose and glucose 6-phosphate to trehalose, homologous to Tps3p and may share function |  | Y | |  | |
| YBR020W | 1.55 | 9.72E-04 | *GAL1* | Galactokinase, phosphorylates alpha-D-galactose to alpha-D-galactose-1-phosphate in the first step of galactose catabolism; expression regulated by Gal4p |  | Y | |  | |
| YDR538W | 1.61 | 1.55E-03 | *PAD1* | Phenylacrylic acid decarboxylase, confers resistance to cinnamic acid, decarboxylates aromatic carboxylic acids to the corresponding vinyl derivatives; homolog of E. coli UbiX |  | Y | |  | |
| YLR258W | 2.77 | 6.34E-03 | *GSY2* | Glycogen synthase, similar to Gsy1p; expression induced by glucose limitation, nitrogen starvation, heat shock, and stationary phase; activity regulated by cAMP-dependent, Snf1p and Pho85p kinases as well as by the Gac1p-Glc7p phosphatase |  | Y | |  | |
| YHL012W | 0.85 | 8.07E-03 | *n/a* | Putative protein of unknown function, has some homology to Ugp1p, which encodes UDP-glucose pyrophosphorylase |  | Y | |  | |
| YFR015C | 2.05 | 7.41E-05 | *GSY1* | Glycogen synthase with similarity to Gsy2p, the more highly expressed yeast homolog; expression induced by glucose limitation, nitrogen starvation, environmental stress, and entry into stationary phase | Y |  | |  | |
| YJL172W | 1.47 | 5.17E-04 | *CPS1* | Vacuolar carboxypeptidase yscS; expression is induced under low-nitrogen conditions | Y |  | |  | |
| YJL166W | 2.99 | 6.09E-04 | *QCR8* | Subunit 8 of ubiquinol cytochrome-c reductase complex, which is a component of the mitochondrial inner membrane electron transport chain; oriented facing the intermembrane space; expression is regulated by Abf1p and Cpf1p | Y |  | |  | |
| YHR216W | 1.36 | 1.08E-03 | *IMD2* | Inosine monophosphate dehydrogenase, catalyzes the first step of GMP biosynthesis, expression is induced by mycophenolic acid resulting in resistance to the drug, expression is repressed by nutrient limitation | Y |  | |  | |
| YAL062W | 1.66 | 3.61E-03 | *GDH3* | NADP(+)-dependent glutamate dehydrogenase, synthesizes glutamate from ammonia and alpha-ketoglutarate; rate of alpha-ketoglutarate utilization differs from Gdh1p; expression regulated by nitrogen and carbon sources | Y |  | |  | |
| YMR101C | 1.74 | 5.89E-03 | *SRT1* | Cis-prenyltransferase involved in synthesis of long-chain dolichols (19-22 isoprene units; as opposed to Rer2p which synthesizes shorter-chain dolichols); localizes to lipid bodies; transcription is induced during stationary phase | Y |  | |  | |
| YJR078W | -1.50 | 4.12E-03 | *BNA2* | Putative tryptophan 2,3-dioxygenase or indoleamine 2,3-dioxygenase, required for the de novo biosynthesis of NAD from tryptophan via kynurenine; expresssion is upregulated upon telomere uncapping; regulated by Hst1p and Aft2p |  | Y | |  | |
| YGR287C | -1.06 | 4.80E-03 | *n/a* | Protein of unknown function that may interact with ribosomes, based on co-purification experiments; has similarity to alpha-D-glucosidase (maltase); authentic, non-tagged protein detected in purified mitochondria in high-throughput studies | Y |  | |  | |
| YKL029C | -1.10 | 4.12E-03 | *MAE1* | Mitochondrial malic enzyme, catalyzes the oxidative decarboxylation of malate to pyruvate, which is a key intermediate in sugar metabolism and a precursor for synthesis of several amino acids | Y |  | |  | |
| YML075C | -1.69 | 3.32E-04 | *HMG1* | One of two isozymes of HMG-CoA reductase that catalyzes the conversion of HMG-CoA to mevalonate, which is a rate-limiting step in sterol biosynthesis; localizes to the nuclear envelope; overproduction induces the formation of karmellae | Y |  | |  | |

**NOTES:** 1. Descriptions adopted from Saccharomyces Genome Database (SGD). *n/a* refers to not available.

| **Table S2.** Transcriptome: S288c v CEN.PK113-7D Galactose (*p_adj_*<0.01) | | | | | | |  |  |
| --- | --- | --- | --- | --- | --- | --- | --- | --- |
| **Systematic Gene Name** | **logFC** | **p_adj_ value** | **Standard Gene Name** | **Description** | **Silent SNP** | **Nonsynonymous SNP** |  | |
| YMR250W | 2.96 | 1.54E-04 | *GAD1* | Glutamate decarboxylase, converts glutamate into gamma-aminobutyric acid (GABA) during glutamate catabolism; involved in response to oxidative stress |  | Y |  | |
| YBR001C | 1.66 | 3.81E-04 | *NTH2* | Putative neutral trehalase, required for thermotolerance and may mediate resistance to other cellular stresses |  | Y |  | |
| YHL012W | 1.36 | 9.38E-04 | *n/a* | Putative protein of unknown function, has some homology to Ugp1p, which encodes UDP-glucose pyrophosphorylase |  | Y |  | |
| YML100W | 2.34 | 1.08E-03 | *TSL1* | Large subunit of trehalose 6-phosphate synthase (Tps1p)/phosphatase (Tps2p) complex, which converts uridine-5'-diphosphoglucose and glucose 6-phosphate to trehalose, homologous to Tps3p and may share function |  | Y |  | |
| YPL268W | 1.27 | 2.52E-03 | *PLC1* | Phospholipase C, hydrolyzes phosphatidylinositol 4,5-biphosphate (PIP2) to generate the signaling molecules inositol 1,4,5-triphosphate (IP3) and 1,2-diacylglycerol (DAG); involved in regulating many cellular processes |  | Y |  | |
| YAL054C | 1.70 | 2.54E-03 | *ACS1* | Acetyl-coA synthetase isoform which, along with Acs2p, is the nuclear source of acetyl-coA for histone acetlyation; expressed during growth on nonfermentable carbon sources and under aerobic conditions |  | Y |  | |
| YKR066C | 1.04 | 3.22E-03 | *CCP1* | Mitochondrial cytochrome-c peroxidase; degrades reactive oxygen species in mitochondria, involved in the response to oxidative stress |  | Y |  | |
| YLR258W | 2.91 | 3.93E-03 | *GSY2* | Glycogen synthase, similar to Gsy1p; expression induced by glucose limitation, nitrogen starvation, heat shock, and stationary phase; activity regulated by cAMP-dependent, Snf1p and Pho85p kinases as well as by the Gac1p-Glc7p phosphatase |  | Y |  | |
| YDR530C | 0.76 | 6.36E-03 | *APA2* | Diadenosine 5',5''-P1,P4-tetraphosphate phosphorylase II (AP4A phosphorylase), involved in catabolism of bis(5'-nucleosidyl) tetraphosphates; has similarity to Apa1p |  | Y |  | |
| YER024W | 2.17 | 8.48E-03 | *YAT2* | Carnitine acetyltransferase; has similarity to Yat1p, which is a carnitine acetyltransferase associated with the mitochondrial outer membrane |  | Y |  | |
| YAL062W | 3.29 | 1.72E-04 | *GDH3* | NADP(+)-dependent glutamate dehydrogenase, synthesizes glutamate from ammonia and alpha-ketoglutarate; rate of alpha-ketoglutarate utilization differs from Gdh1p; expression regulated by nitrogen and carbon sources | Y |  |  | |
| YFR015C | 1.44 | 3.21E-04 | *GSY1* | Glycogen synthase with similarity to Gsy2p, the more highly expressed yeast homolog; expression induced by glucose limitation, nitrogen starvation, environmental stress, and entry into stationary phase | Y |  |  | |
| YJL166W | 3.16 | 4.40E-04 | *QCR8* | Subunit 8 of ubiquinol cytochrome-c reductase complex, which is a component of the mitochondrial inner membrane electron transport chain; oriented facing the intermembrane space; expression is regulated by Abf1p and Cpf1p | Y |  |  | |
| YER065C | 2.71 | 4.85E-04 | *ICL1* | Isocitrate lyase, catalyzes the formation of succinate and glyoxylate from isocitrate, a key reaction of the glyoxylate cycle; expression of ICL1 is induced by growth on ethanol and repressed by growth on glucose | Y |  |  | |
| YDR058C | 2.16 | 3.19E-03 | *TGL2* | Protein with lipolytic activity towards triacylglycerols and diacylglycerols when expressed in E. coli; role in yeast lipid degradation is unclear | Y |  |  | |
| YHR216W | 0.89 | 5.40E-03 | *IMD2* | Inosine monophosphate dehydrogenase, catalyzes the first step of GMP biosynthesis, expression is induced by mycophenolic acid resulting in resistance to the drug, expression is repressed by nutrient limitation | Y |  |  | |
| YHR018C | 1.37 | 9.04E-03 | *ARG4* | Argininosuccinate lyase, catalyzes the final step in the arginine biosynthesis pathway | Y |  |  | |
| YGR287C | -1.72 | 4.97E-04 | *n/a* | Protein of unknown function that may interact with ribosomes, based on co-purification experiments; has similarity to alpha-D-glucosidase (maltase); authentic, non-tagged protein detected in purified mitochondria in high-throughput studies | Y |  |  | |
| YJL172W | -1.04 | 1.95E-03 | *CPS1* | Vacuolar carboxypeptidase yscS; expression is induced under low-nitrogen conditions | Y |  |  | |
| YKL029C | -1.07 | 3.55E-03 | *MAE1* | Mitochondrial malic enzyme, catalyzes the oxidative decarboxylation of malate to pyruvate, which is a key intermediate in sugar metabolism and a precursor for synthesis of several amino acids | Y |  |  | |
| YML075C | -0.74 | 8.58E-03 | *HMG1* | One of two isozymes of HMG-CoA reductase that catalyzes the conversion of HMG-CoA to mevalonate, which is a rate-limiting step in sterol biosynthesis; localizes to the nuclear envelope; overproduction induces the formation of karmellae | Y |  |  | |

**NOTES:** 1. Descriptions adopted from *Saccharomyces* Genome Database (SGD). *n/a* refers to not available.

**Table S3**. Description of *S. cerevisiae* strains

| **Strain Name** | **Genotype** | **Source** | **References** |
| --- | --- | --- | --- |
| S288c | *MAT*α *SUC2 gal2 mal mel flo1 flo8-1 hap1 ho bio1 bio6* | ATCC®^A^ | Mortimer, 1986  Johnston, 1994  Goffeau 1996  Cherry, 1997 |
|  | First *S. cerevisiae* sequenced and stored in SGD. Strain not capable of anaerobic galactose metabolism (*gal2*), and contains a mutated copy of *HAP1* with a Ty1 insertion in the carboxy terminus. Strain resequenced as part of SGRP. Strain is a prototrophic haploid. |
| CEN.PK113-7D | *MATa URA3 HIS3 LEU2 TRP1 SUC2 MAL2-8^C^* | SRD GmbH^B^ | Cherry, 1997  van Dijken, 2000 |
|  | CEN.PK strain family was constructed as part of an interdisciplinary German research project ("Stofflüsse in Mikroorganissmen"). Strain is a prototrophic haploid. The strains were obtained from Dr. P. Kötter (J.W. Universität, Frankfurt, Germany). |
| **NOTES:** | A. American Type Culture Collection (ATCC®). |  |  |
|  | B. Scientific Research and Development (SRD) GmbH. |  |  |

**Table S4. EDENA determination for *de novo* assembly of S288c and CEN.PK113-7D sequences**

| **Genome Sequencing Parameter** |  | **S288c** | **CEN.PK113-7D** |
| --- | --- | --- | --- |
| *EDENA Software Determination for de novo Assembly (Secondary Check of SNP Detection)* |  |  |  |
| Reads Base Length (bp) |  | 35 | 35 |
| No. of Reads |  | 5,301,907 | 6,603,200 |
| No. of Unique Reads (Aligned) |  | 4,387,286 | 5,045,108 |
| No. of Contigs |  | 12,775 | 16,436 |
| Total Base Length (bp) |  | 4,440,488 | 7,326,814 |
| Average Contig Length (bp) |  | 345 | 446 |
| Maximum Contig Length (bp) |  | 2,031 | 2,734 |
| Genome Reference Coverage (%) |  | 36 | 59.9 |
| Total No. of SNPs Detected |  | 696 | 13,984 |
|  |  |  |  |
|  |  |  |  |
| **Metabolic SNP Detection** |  | **S288c** | **CEN.PK113-7D** |
| *EDENA Software Determination for de novo Assembly (Secondary Check of SNP Detection)* |  |  |  |
| Reads Base Length (bp) |  | 35 | 35 |
| No. of Reads |  | 5,301,907 | 6,603,200 |
| No. of Unique Reads (Aligned) |  | 4,387,286 | 5,054,108 |
| No. of Contigs |  | 12,775 | 16,436 |
| Total Base Length (bp) |  | 4,400,488 | 7,326,814 |
| Average Contig Length (bp) |  | 345 | 446 |
| Maximum Contig Length (bp) |  | 2,031 | 2,734 |
| Genome Reference Coverage (%) |  | 41.7 | 65.7 |
| Total No. of SNPs Detected |  | 71 | 1133 |
